# Synoviocytes protect cartilage from the effects of injury in vitro

**DOI:** 10.1186/1471-2474-14-54

**Published:** 2013-02-01

**Authors:** Christina M Lee, John D Kisiday, C Wayne McIlwraith, Alan J Grodzinsky, David D Frisbie

**Affiliations:** 1 Orthopaedic Research Center, Department of Clinical Sciences, College of Veterinary Medicine, Colorado State University, 300 West Drake Rd., Fort Collins, Colorado CO 80523, USA; 2 Biomedical Engineering, Department of Biological Engineering, Massachusetts Institute of Technology, 77 Massachusetts Ave, Cambridge, MA, 02139, USA

**Keywords:** Cartilage, Synovial cell, Injury

## Abstract

**Background:**

It is well documented that osteoarthritis (OA) can develop following traumatic joint injury and is the leading cause of lameness and subsequent wastage of equine athletes. Although much research of injury induced OA has focused on cartilage, OA is a disease that affects the whole joint organ.

**Methods:**

In this study, we investigated the impact of synovial cells on the progression of an OA phenotype in injured articular cartilage. Injured and control cartilage were cultured in the presence of synoviocytes extracted from normal equine synovium. Synoviocytes and cartilage were evaluated for catabolic and anabolic gene expression. The cartilage was also evaluated histologically for loss of extracellular matrix molecules, chondrocyte cell death and chondrocyte cluster formation.

**Results:**

The results indicate synoviocytes exert both positive and negative effects on injured cartilage, but ultimately protect injured cartilage from progressing toward an OA phenotype. Synoviocytes cultured in the presence of injured cartilage had significantly reduced expression of aggrecanase 1 and 2 (ADAMTS4 and 5), but also had increased expression of matrix metalloproteinase (MMP) -1 and reduced expression of tissue inhibitor of metalloproteinases 1 (TIMP-1). Injured cartilage cultured with synoviocytes had increased expression of both collagen type 2 and aggrecanase 2. Histologic examination of cartilage indicated that there was a protective effect of synoviocytes on injured cartilage by reducing the incidence of both focal cell loss and chondrocyte cluster formation, two major hallmarks of OA.

**Conclusions:**

These results support the importance of evaluating more than one synovial joint tissue when investigating injury induced OA.

## Background

Osteoarthritis (OA) is the most common musculoskeletal disease in humans, and is the most common joint disease in horses [1]. Although OA is not a disease that exclusively affects the articular cartilage, the critical criteria are the degradation and eventual loss of cartilage. In addition to the articular cartilage the synovial membrane, fibrous joint capsule and subchondral bone are also compromised in an osteoarthritic joint. Synovial inflammation has been detected in both early and late OA in humans [2-5] and in an equine in vivo study, synovial inflammation alone, without injury or joint instability, was sufficient to induce degradation of articular cartilage [6]. The synovial fluid of injured or diseased joints has been shown to contain anabolic and catabolic cytokines such as prostaglandin E_2_ (PGE_2_), Interleukin (IL) -1β, IL-6, IL-10, IL-1RA, tumor necrosis factor alpha (TNF-α), transforming growth factor beta (TGFβ), and fibroblast growth factor 2 (FGF2), as well as matrix degrading enzymes including aggrecanases (ADAMTS4 and 5), and matrix metalloproteinase (MMP) MMP-1 [7-11]. Both Il-1 and TNF-α have also been shown to induce chondrocyte expression of MMPs by an autocrine/paracrine mechanism [12] and promote enhanced proteoglycan loss in injured cartilage samples [13]. It has even been suggested that synovial inflammation may promote altered chondrocyte gene expression to favor catabolic activities and extracellular matrix (ECM) destruction [14,15].

Although previous studies investigating the relationship between the synovium and cartilage are limited, intercellular interactions have been identified where synovial cells and chondrocytes communicate by direct contact [16] or indirectly by synovial cell release of molecular agents [17]. Additionally, the presence of synovial tissue has been shown to alter the progression of IL-1β induced OA in cartilage explants in vitro [18] and to inhibit cartilage biosynthesis in cartilage explants by an IL-1 independent pathway [19], both suggesting that the progression of ECM degradation and OA is dependent on both the cartilage and the synovium. In a more recent in vitro model the co-culture of injured cartilage with joint capsule explants enhanced the deleterious effects of injury on catabolic gene expression in cartilage and resulted in a reduction of cartilage aggrecan content [20]. Therefore in this study we sought to investigate how co-culture of cartilage and synovial cells (synoviocytes) affects chondrocyte and synoviocyte gene expression profiles as well as cartilage pathology after cartilage injury. In this article we demonstrate that the culture of synoviocytes in the presence of injured cartilage reduces the presence of markers of early OA in cartilage such as focal cell loss and chondrocyte cluster formation, and alters chondrocyte gene expression to favor anabolic activity.

## Methods

### Isolation of cartilage plugs

Equine cartilage samples were extracted from cadaveric stifle joints within sixteen hours of death from 12 different horses (ages 3–5 years) that died for reasons unrelated to the musculoskeletal system and factors not influencing this study. Using aseptic technique (sterile gloves, instruments and media) osteochondral plugs 5 mm in diameter (varying heights) were harvested from the trochlear ridges of the left and right limbs using a customized cylindrical osteotome (Sontec Instruments, Centennial CO). Due to the non-uniform swelling of the cartilage that occurs during culture when the cartilage remains attached to the subchondral bone, the cartilage was removed from the subchondral bone at the calcified and non-calcified cartilage junction immediately after extraction from the joint. To allow for recovery of the chondrocytes from tissue harvest the explants were cultured for forty-eight hours in full growth medium (DMEM (Invitrogen, Carlsbad, CA), 10% fetal bovine serum (v/v) (Hyclone, Logan, UT), 20 μg/ml ascorbic acid (Sigma Aldrich, St Louis, MO), 1% penicillin and streptomycin (v/v) (Invitrogen, Carlsbad, CA), 10 mM HEPES (Invitrogen, Carlsbad, CA), 0.1 mM non-essential amino acids (Invitrogen, Carlsbad, CA), 0.4 mM proline (Sigma Aldrich, St Louis, MO), 0.25 μg/ml amphotericin B (Invitrogen, Carlsbad, CA)) prior to commencement of the experiments.

### Synoviocyte isolation

**Figure 1:**
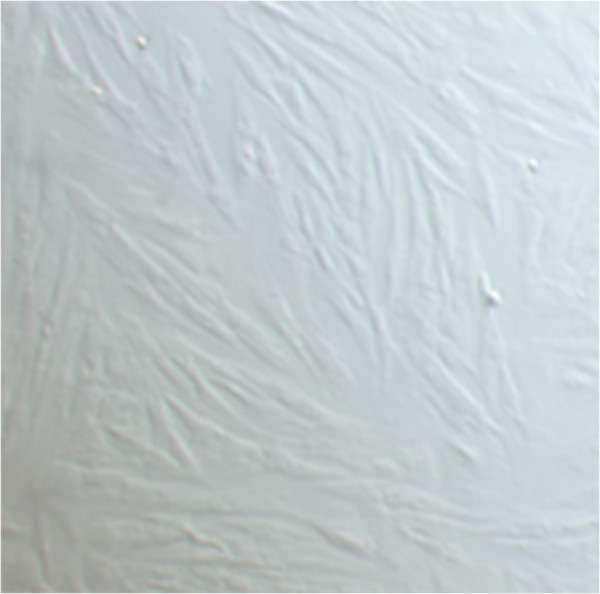
**Fibroblastic synoviocyte phenotype. **Digital Image of synoviocytes harvested from a horse and expanded to passage 3. Image obtained at 10x magnification.

Synoviocytes were isolated from synovial tissue harvested from the stifle of 6 different horses than those used for cartilage harvest (ages 3–5) that also died for reasons unrelated to the musculoskeletal system and to this study. Synovial tissue was minced and incubated with media containing collagenase type II (Worthington Biochemical, Lakewood, NJ) in a spin column flask for 4 hours. Synoviocytes were then filtered from the media using a 40 μm filter and culture expanded in full growth medium (Ham’s F12(Invitrogen, Carlsbad, CA), 10% FBS (v/v) (Hyclone, Logan, UT), 1% antibiotic and antimycotic (v/v) (Invitrogen, Carlsbad, CA) and 10 mM HEPES (Invitrogen, Carlsbad, CA)) to passage 3 to ensure >95% of the cells will express a fibroblastic phenotype (Figure 1) [21] with minimal presence of lymphocytes, natural killer cells and macrophages [22] followed by cryopreservation until experiment commencement.

### Mechanical injury

Just prior to injury, the thickness of each cartilage plug (plane perpendicular to the articular surface; average thickness 1.33 mm) was measured using digital calipers. Cartilage plugs were injured as previously defined [23]. In brief, cartilage plugs were removed from media and placed into a sterile polysulphone loading chamber consisting of a well aligned coaxially with an impermeable platen (10 mm diameter) in the absence of media. Using a H1KS benchtop universal testing machine (Tinius Olsen, Horcham PA) an initial compressive tare load of 4 N was applied. After creep equilibrium was attained for 5 seconds, an injurious compression at a rate of 100% strain/second was applied until 60% final cartilage strain was achieved. Compression was then released at the same rate and plugs were immediately placed into the appropriate culture condition.

### Cartilage-synoviocyte co-culture

Synoviocyte and cartilage were co-cultured using a transwell plate with a 0.4 μm microporous insert (Corning Inc., Corning, NY). Cartilage and synoviocytes were randomly paired for co-culture to form 2 sets of experiments. One set of experiments involved the culture of synoviocytes with cartilage for gene expression analysis at 1, 2, 4 and 8 days in co-culture. The other set of experiments involved the culture of synoviocytes with cartilage for histologic and immunologic evaluation at days 8, 16 or 32 in culture. The time points to evaluate gene expression were determined based on previous studies that have evaluated changes in gene expression in cartilage after injury [18,20]. For histologic evaluation we extended the duration of culture because in previous studies [23] we found injured cartilage required culture for at least 28 days to develop histologic changes. Each experiment was conducted in duplicate, and all experiments repeated a total of 6 times using cartilage from a new horse for each experiment and synoviocytes from 6 different horses total; synoviocytes from each horse were used for one gene expression and one histology experiment. Each experiment consisted of four conditions; injured and control (uninjured) cartilage cultured alone or with synoviocytes. Experiments for gene expression analysis included the fifth condition of synoviocytes cultured alone. Forty-eight hours prior to commencement of experiment synoviocytes were recovered from liquid nitrogen and seeded into the bottom well of a 24-well transwell system at a seeding density of 5000 cells/cm^2^ and cultured in growth media composed of half cartilage DMEM media and half synoviocyte F12 media. Immediately after injury, cartilage plugs were added to the top well of the transwell system, uninjured control cartilage samples were maintained in parallel. All samples remained in culture for 1, 2, 4, 8, 16 or 32 days, with media changes every 2–3 days.

### Sample collection

For gene expression analysis, cartilage and synoviocytes were removed from incubation at 1, 2, 4 and 8 days and immediately placed on ice. The cartilage plugs were rinsed with ice cold RNase free PBS then snap frozen in Trizol (Invitrogen, Carlsbad, CA) and stored at −80°C. Similarly the synoviocytes were rinsed with ice cold RNase free PBS, treated with 350 ul of RLT lysis buffer (RNeasy minikit, Qiagen, Valencia CA) and stored in −80°C. For histology and immunohistochemistry (IHC) cartilage samples at days 8, 16 and 32 were removed from culture and sectioned across the diameter perpendicular to the fissures when present (arbitrarily in the middle of the sample when not) and always along a plane perpendicular to the superficial surface. One half of each sample was placed into 10% neutral buffered formalin for histologic analysis and the remaining half was embedded in OCT, snap frozen in liquid nitrogen and stored at −80°C for immunohistochemistry.

### RNA extraction

To extract RNA from synoviocytes, samples in RLT lysis buffer were thawed and passed through a QIAshredder homogenization column (Qiagen, Valencia, CA) followed by RNA extraction using the RNeasy Mini Kit (Qiagen, Valencia, CA) according to the manufacturer’s instructions with on column genomic DNA digest using RNase free DNase (Qiagen, Valencia, CA). To extract RNA from cartilage, the plugs were pulverized and homogenized in Trizol (Invitrogen, Carlsbad, CA) on ice using a tissue homogenizer. The homogenized sample was then allowed to incubate at room temperature for 20 minutes in Trizol followed by centrifugation at 12,000 x g for 12 minutes at 4°C. The supernatant was transferred to a new microcentrifuge tube, mixed with 400 ul chloroform, incubated at room temperature for 3 minutes and then centrifuged at 12,000 × g for 15 minutes at 4°C. The aqueous phase was transferred to a new microcentrifuge tube, mixed with 200 ul chloroform, incubated at room temperature for 3 minutes then centrifuged at 12,000 × g for 15 minutes at 4°C. The aqueous phase was transferred to a new microcentrifuge tube to which 500 ul isopropanol was added. The samples were incubated at room temperature for 10 minutes then centrifuged at 12,000 × g for 10 minutes at 4°C to pellet the RNA. The RNA fraction was cleaned with 70% ethanol and re-suspended with RNase free water followed by digestion of genomic DNA using RNase free DNase (Qiagen, Valencia, CA).

### Real time PCR

Synoviocyte mRNA was evaluated for expression of cyclooxygenase 2 (cox-2), interleukin 1β, 6, 10 (IL-1β, -6, -10), interleukin 1 receptor antagonist protein (IRAP), matrix metalloproteinase 1, 3, 13 (MMP-1,-3,-13), transforming growth factor β (TGF-β), tissue inhibitor of metalloproteinases 1 (TIMP-1), aggrecanase 1, 2 (ADAMTS4, 5) and fibroblast growth factor 2 (FGF2). Chondrocyte mRNA was evaluated for collagen types 1 and 2 (Col1 and Col2), aggrecan, IL-1β, -6, -10, TNF-α, TGF-β, MMP-1, -3, -13, ADAMTS4, 5 and TIMP-1. All expression levels were normalized to glyceraldehyde 3-phosphate dehydrogenase (GAPDH) as screening of all samples determined no variability in GAPDH expression. Equal concentrations of RNA (5 ng) from each sample were reverse transcribed into cDNA using superscript First-Strand Synthesis for RT-PCR (Invitrogen, Carlsbad, CA). Relative gene expression levels were determined by semi-quantitative real time PCR using TaqMan based probes and primers purchased from the Lucy Whittier Molecular and Diagnostic core facility at University of California, Davis, or from the Orthopaedic Research Center at Colorado State University, Fort Collins (Table 1). All real time PCR assays were run on the ABI Prism 7000 Sequence Detection System (Applied Biosystems, Forster City, CA). Relative gene expression (using the delta delta Ct method [24]) was determined by comparing gene expression levels to baseline, where baseline for synoviocytes is equal to expression in synoviocytes cultured alone, and for injured cartilage cultured alone or in co-culture baseline is equal to control cartilage cultured alone or in co-culture respectively.

**Table 1:** Table of primer and probe sequences from the Orthopaedic Research Center

**Gene**	**Forward**	**Reversec**	**TaqMan probe**
IL-1B	AGTCTTCAGTGCTCAGGTTTCTGA	TGCCGCTGCAGTAAGTCATC	CAGCCATGGCAGCAGTACCCGA
IL-10	TTCAGCAGGGTGAAGACTTTC	CTTGGCAACCCAGGTAACCCTTA	TGTTGAACGGGTCCCTGCTGGAG
TGF-B	GTTAAGCGTGGAGCAGCAT	AGTGACATCAAAGGACAGCC	CTGCTGACCCCCAGCGACTCG
ADAMTS4	TGTGATCGTGTCATTGGCTCC	TGTTTGCTGCAGCTAGAACCATC	AGTTTGACAAGTGCATGGTGTGCGGT
ADAMTS5	AAGGTGACTGATGGGACCGAATGT	TTTGAGCCAATGATGCCGTCACAG	AGGCCATACAGTAATTCCGTCTGCGT

### Histology

Formalin fixed samples were paraffin embedded, and sectioned (5 μm thick) using a Leica RM2255 microtome (Leica Biosystems, Buffalo Grove IL). One slide each from an injured and control sample was stained with Hematoxylin and Eosin (H&E) to evaluate cellular pathologic changes or Safranin O Fast Green (SOFG) to detect changes in regional glycosaminoglycan (GAG) content. All images of slides were obtained using Q-capture (QImaging, Surrey BC).

### Immunohistochemistry (IHC)

Immunohistochemical staining was conducted for the detection of aggrecan and collagen type II. Briefly, frozen embedded samples were sectioned (8 μm thick) and mounted onto adhesive slides using the CryoJane® Tape-Transfer system (Instrumedics Inc., St Louis, MO). Sections were probed with mouse antibodies raised against aggrecan at a 1:20 dilution (Novus Biologics, Littleton, CO) or collagen type II using undiluted supernatant (Hybridoma Bank, Iowa City, IA), followed by probing with a goat anti mouse secondary antibody conjugated with horseradish peroxidase at a 1:500 dilution (Jackson Immunoresearch West Grove, PA) and antibody detection with VECTOR® NovaRED™ (Vector laboratories, Burlingame, CA). For negative controls, additional sections were probed with normal mouse serum at a concentration equal to that of the primary antibody.

### Section grading

All histological and IHC sections were blindly evaluated using a previously established grading scale detailing the severity of OA characteristics in equine cartilage [25]. In brief, each region was assigned a grade for each OA characteristic that corresponds to the severity by which the sample deviates in histologic appearance compared to normal articular cartilage where 0 = normal or no change, 1 = slight, 2 = mild, 3 = moderate. To eliminate the inclusion or artifact from tissue harvest in the analysis, the outer 5% of the tissue around the left and right edges and below the deep zone were not graded. One section was graded for each sample. For each OA characteristic, scores for each region (superficial, middle, deep and fissures) were evaluated separately then summed together for each slide, the maximum score for each of the 4 regions was 3 creating a maximum score for all regions combined of 12. Sections stained with H&E were used to visualize generalized chondrocyte cell death (determined by the amount of lacunae lacking discernable nuclei), localized cell death/focal cell loss (the presence of distinct regions devoid of discernable nuclei) and chondrocyte cluster formation (lacunae containing more than one nuclei). Changes to the ECM were determined using both histologic and IHC stained sections. Safranin O Fast Green stained sections were used to identify proteoglycan content of control and treated cartilage. To identify ECM molecules more specifically, sections stained by IHC were used to assess regions containing aggrecan and collagen type II in control and treated cartilage.

### Statistical analysis

Statistical analysis was conducted to determine differences in IHC and histologic staining results accounting for both injury and location using Proc GLIMMIX. Predictive F-values were used to determine statistical differences between injury and control or between regions, with specific comparisons made using a least squares means procedure, both with significance set at α = 0.05.

## Results

### Gene expression

**Figure 2:**
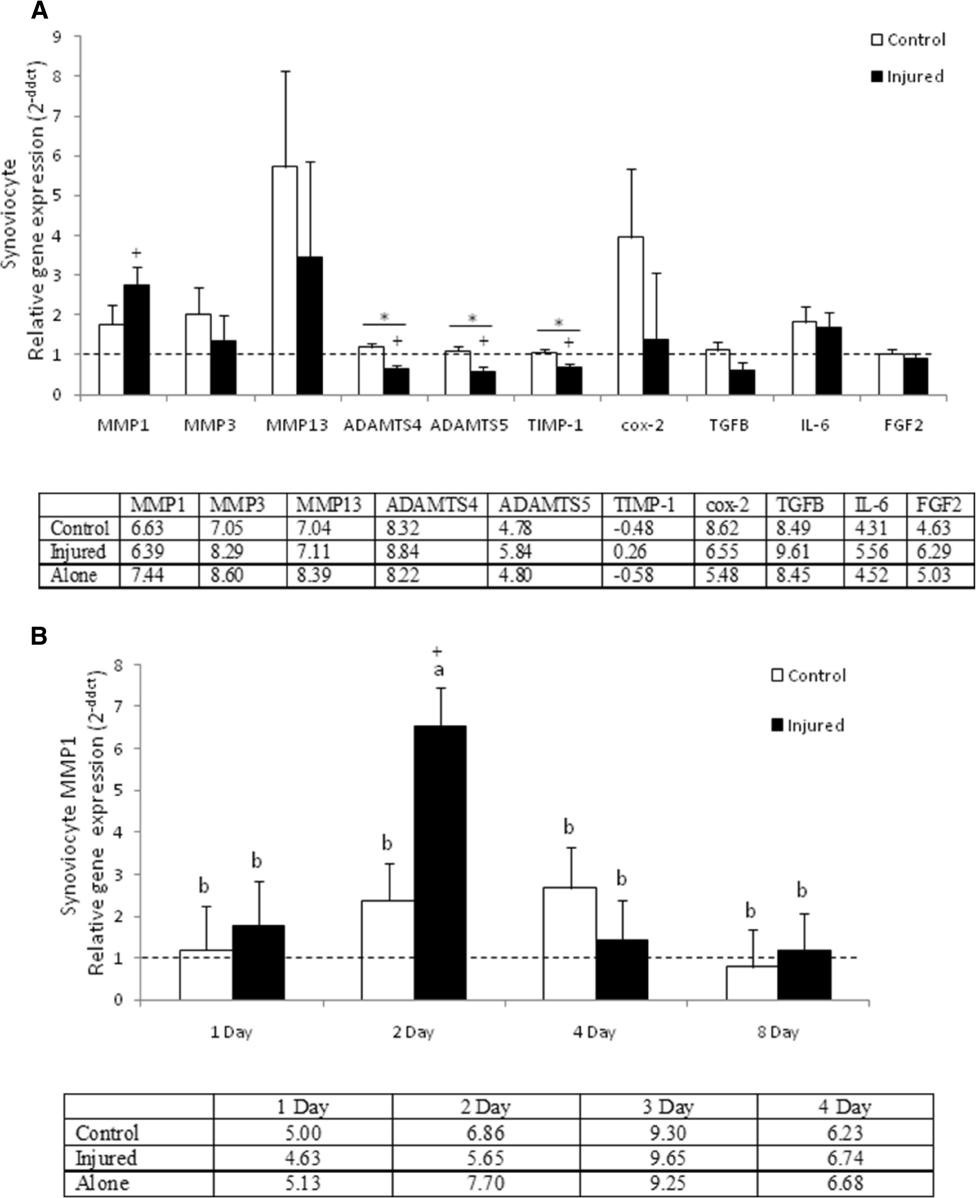
**Synoviocyte gene expression. **(**A**) Gene expression data for synoviocytes in the presence of injured or control cartilage. Data presented as mean relative expression across all time points for each culture condition (± SEM) * denotes statistically significant difference between pairs, + denotes statistically significant difference from baseline, p-value < 0.05. (**B**) MMP-1 expression was significantly affected by both treatment and duration in culture with a significant increase in expression in synoviocytes cultured with injured cartilage after 48 hours. Synoviocyte gene expression levels are relative to synoviocytes cultured alone. Bars represent mean (± SEM), bars with the same letter for each pathology are not significantly different from each other, significance set at alpha (p-value) = 0.05, n = 6. Main effects or interactions were evaluated in the AOV table with a predictive F-value (Pr > F).

In synoviocyte samples, mRNA expression of IL-1β, IL-1RA, IL-10 and TNF-α was not detected. There were no significant differences in the expression of cox-2, MMP-3,-13, IL-6, FGF2 and TGFB in synoviocytes cultured with control versus injured cartilage samples (Figure 2A). Synoviocyte expression of MMP-1 (p = 0.0334), ADAMTS4 and 5 (p = 0.0004 and p = 0.0011), and TIMP-1 (p = 0.0010) were significantly affected by culture condition. Synoviocytes cultured in the presence of control cartilage had a significantly higher relative expression of ADAMTS4 and 5 (p < 0.0001 and p = 0.0005) and TIMP-1 (P = 0.0007) compared to synoviocytes cultured with injured cartilage (Figure 2A). Synoviocytes cultured with injured cartilage had significantly higher expression of MMP-1 (p = 0.0095) and significantly lower expression of ADAMTS4 and 5 (p = 0.007 and p = 0.0037) and TIMP-1 (p = 0.0022) compared to baseline synoviocyte culture (Figure 2A). Expression of MMP-1 in synoviocytes was significantly affected by both main effects treatment and duration in culture where synoviocytes cultured in the presence of injured cartilage had significantly greater expression at two days in culture compared to synoviocytes cultured with injured cartilage (p = 0.0339) including baseline (synoviocytes cultured alone) (p < 0.0001) (Figure 2B).

**Figure 3:**
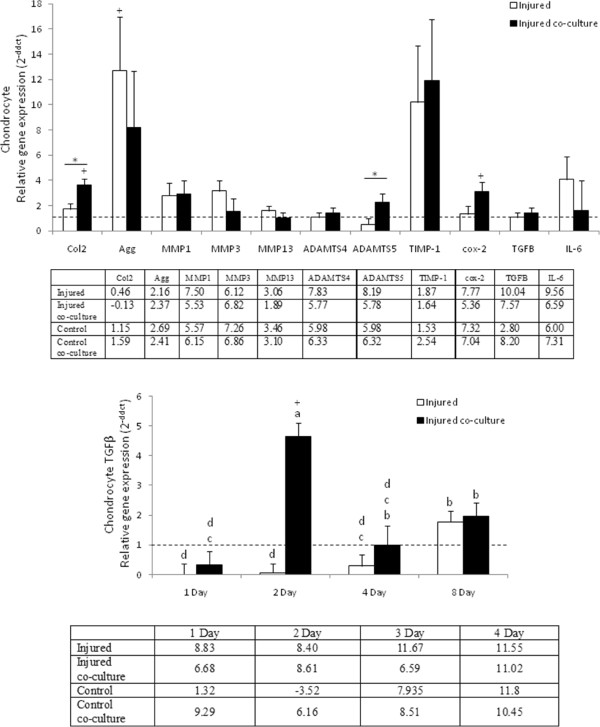
**Chondrocyte gene expression. **(**A**) Gene expression data for injured cartilage cultured alone or with synoviocytes. Data presented as mean relative expression across all time points for each culture condition (± SEM) * denotes statistically significant difference between pairs, + denotes statistically significant difference from baseline p-value < 0.05. (**B**) TGF-β expression was significantly affected by both treatment and duration in culture with a significant increase in expression in synoviocytes cultured with injured cartilage after 48 hours. Chondrocyte gene expression levels for injured cartilage are relative to expression of uninjured cartilage. Bars represent mean (± SEM), bars with the same letter for each pathology are not significantly different from each other, significance set at alpha (p-value) = 0.05, n = 6. Main effects or interactions were evaluated in the AOV table with a predictive F-value (Pr > F).

In cartilage samples we were unable to detect mRNA expression of IL-1β, IL-1RA, IL-10, TNF-α, FGF2 or Col1, and there were no significant differences in expression levels of MMP-1, -3, -13, ADAMTS4, TGFβ and IL-6 between cartilage treatments or compared to baseline (Figure 3A). Injured cartilage co-cultured with synoviocytes had significantly higher expression of collagen type 2 (p = 0.0027) and ADAMTS5 (p = 0.0401) compared to injured cartilage cultured alone (Figure 3A) and had significantly higher expression of type 2 collagen (p <0.0001) and cox-2 (p = 0.0283) compared to control cartilage cultured with synoviocytes (Figure 3A). Injured cartilage cultured alone had significantly higher expression of aggrecan compared to control cartilage (p = 0.0371) (Figure 3A). TGF-β levels were significantly highest in injured cartilage cultured in the presence of synoviocytes at day two compared to all other injured cartilage samples (p <0.0001) and control cartilage cultured with synoviocytes (p <0.0001) (Figure 3B).

### Histologic evaluation

**Figure 4:**
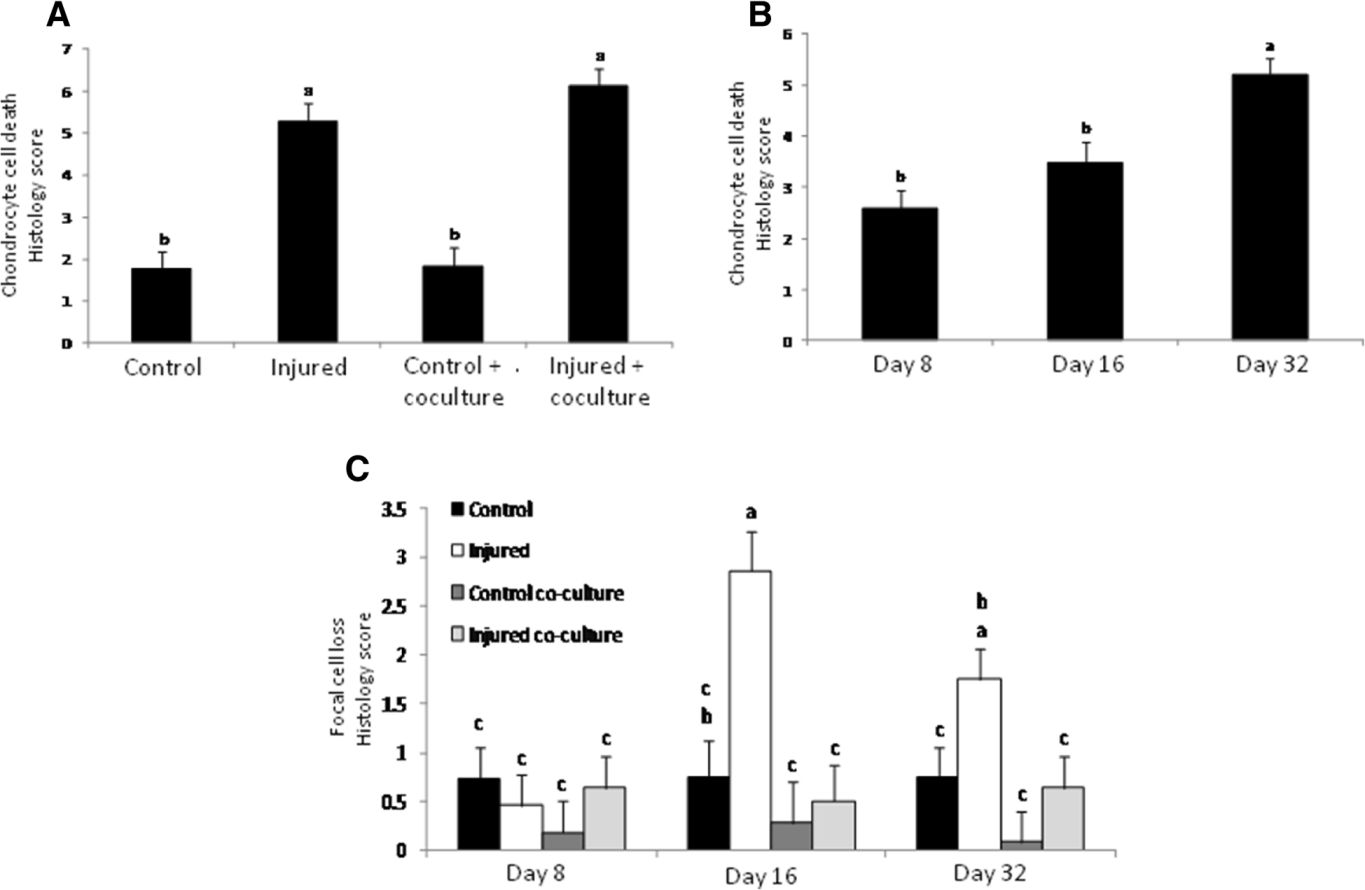
**Chondrocyte cell death and focal cell loss. **(**A**) Chondrocyte cell death was significantly increased in injured cartilage regardless of the presence of synoviocytes and (**B**) independently of treatment was significantly increased at day 32 compared to the earlier time points. (**C**) Focal cell loss was significantly affected by both treatment and days in culture with injured cartilage not in the presence of synoviocytes have the greatest severity at 16 and 32 days compared to the other treatments. Bars represent mean (± SEM), bars with the same letter for each pathology are not significantly different from each other, significance set at alpha (p-value) = 0.05. Main effects or interactions were evaluated in the AOV table with a predictive F-value (Pr > F), n = 6.

**Figure 5:**
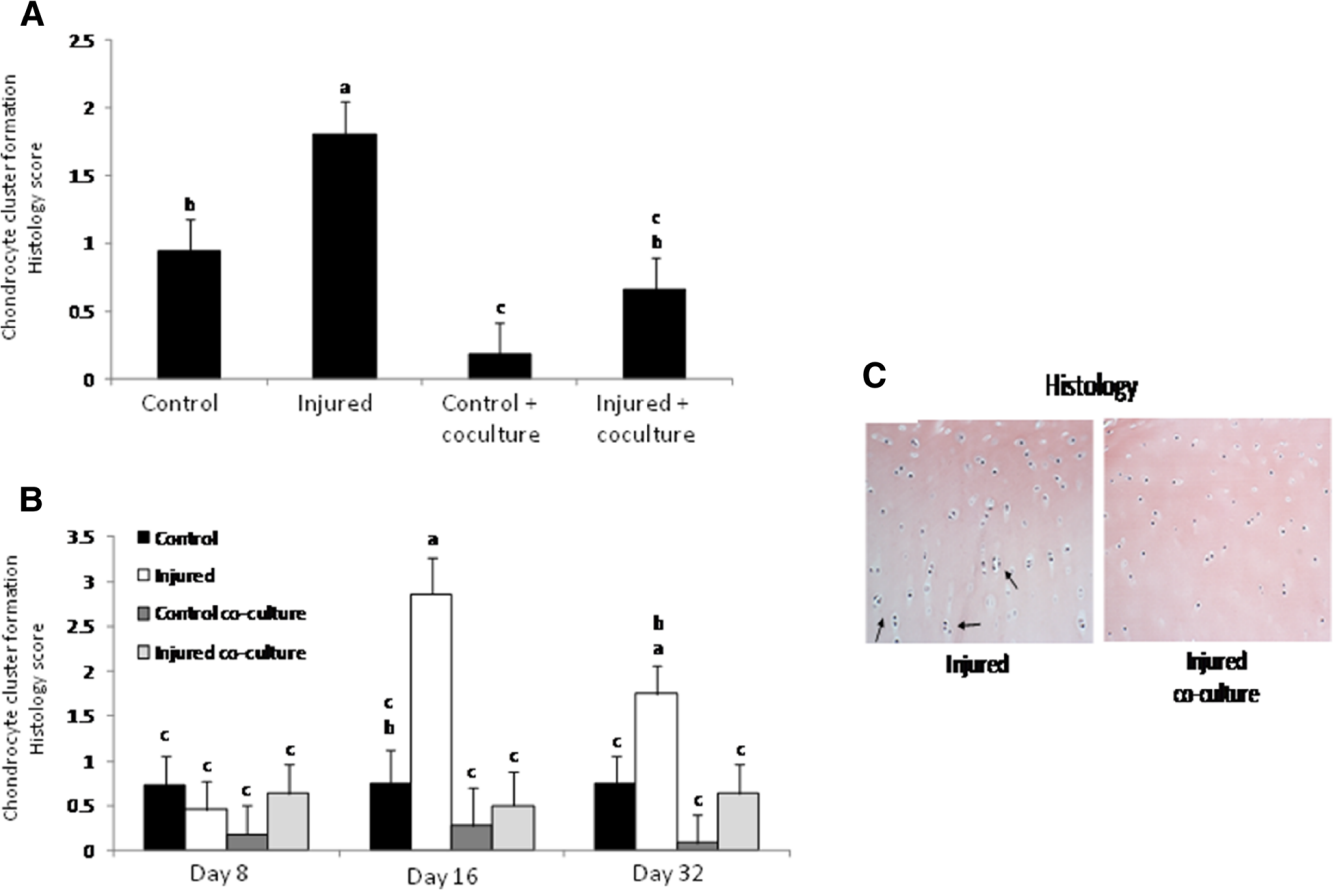
**Chondrocyte cluster formation. **(**A**) The severity of chondrocyte cluster formation was significantly higher in the injured cartilage cultured in the absence of synoviocytes compared to all other treatments. (**B**) The size of chondrocyte clusters was significantly affected by both treatment and duration in culture; injured cartilage samples cultured alone had significantly larger chondrocyte clusters at days 16 and 32 compared to samples in other treatment groups. Bars represent mean (± SEM), bars with the same letter for each pathology are not significantly different from each other, significance set at alpha (p-value) = 0.05. Main effects or interactions were evaluated in the AOV table with a predictive F-value (Pr > F). (**C**) Representative H&E sections showing differences in chondrocyte cluster formation. Picture oriented with superficial surface on top and deep zone on bottom. Arrows indicate chondrocyte clusters, n = 6.

Histologic sections stained with H&E showed increased chondrocyte cell death in injured cartilage cultured with or without synoviocytes compared to both uninjured controls (p < 0.0001). There was no significant difference in generalized cell death between injured cartilage samples cultured with and without synoviocytes (Figure 4A). Independent of treatment, the incidence of cell death in all cartilage samples was significantly increased at day 32 compared to days 8 and 16 (p < 0.0001) (Figure 4B). Injured cartilage cultured alone had a significantly higher incidence of focal cell loss at day 16 and 32 compared to all other treatment groups (p = 0.0258) (Figure 4C). Chondrocyte cluster formation was significantly more severe in injured cartilage cultured alone compared to all other treatments (p < 0.0001) (Figure 5A). The size of chondrocyte clusters (based on number of nuclei) was significantly affected by both treatment and duration in culture. Chondrocyte clusters in the injured cartilage cultured alone were significantly larger at days 16 and 32 compared to the clusters formed in any other treatment (p = 0.0130) (Figure 5B, C).

**Figure 6:**
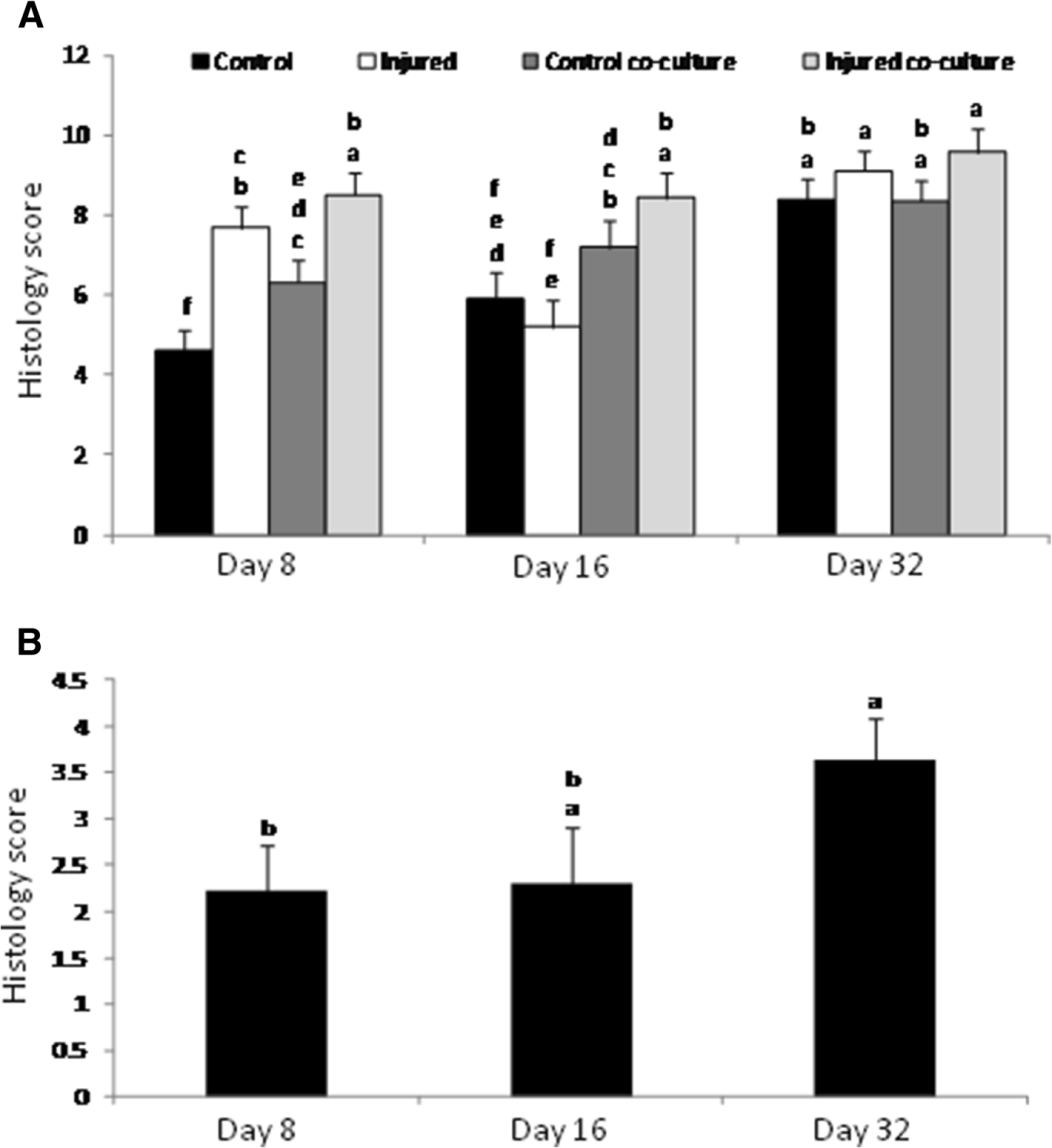
**Changes in extracellular matrix composition determined by histology and IHC. **(**A**) Total proteoglycan content was significantly affected by both treatment and duration in culture. (**B**) Independent of treatment, collagen type II content was significantly affected by duration in culture and was significantly reduced at day 32 compared to day 8. Bars represent mean (± SEM), bars with the same letter for each pathology are not significantly different from each other, significance set at alpha (p-value) = 0.05, n = 6. Main effects or interactions were evaluated in the AOV table with a predictive F-value (Pr > F).

Sections stained with SOFG revealed effects of both treatment and duration in culture (p = 0.0058) (Figure 6A). At day 8, there was a significant reduction in total SOFG staining in all treatment groups compared to control cartilage cultured alone (Figure 6A). At day 16 there was a trend of reduced SOFG staining in samples cultured in the presence of synoviocytes, and at day 32 there was no significant difference in SOFG staining between any treatment (Figure 6A).

**Figure 7:**
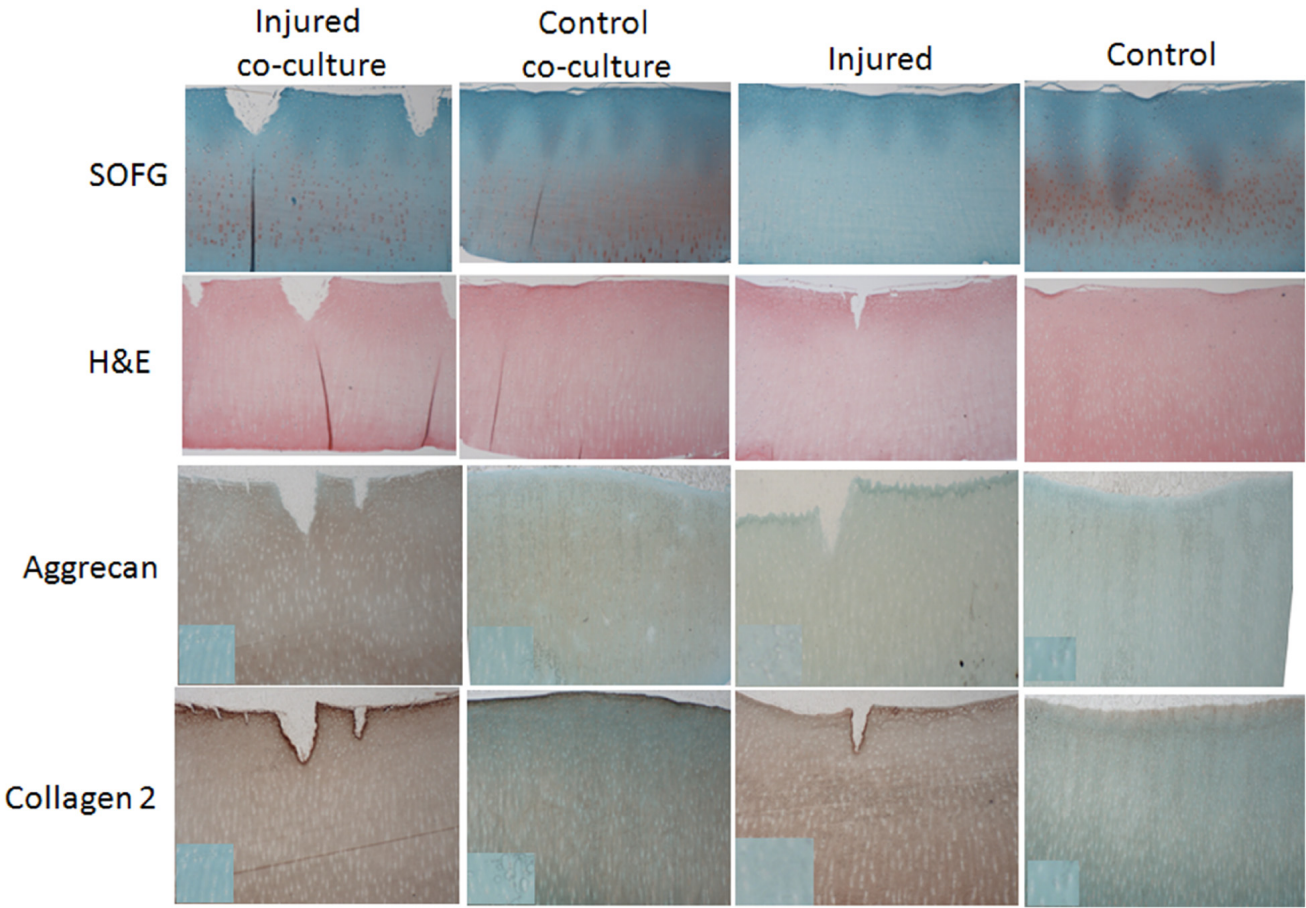
**Representative images of cartilage samples in each culture condition after 32 days in culture. **Images represent slides stained with SOFG and H&E or by immunohistochemistry to detect collagen type II and aggrecan. Box inserted into the images showing collagen type II and aggrecan show the negative control slides.

The results from IHC staining show no significant differences in aggrecan content between any samples (Figure 7). Immunohistochemical staining for collagen type II content indicate collagen was not affected by treatment, however duration in culture had a significant impact with day 32 samples having the most severe reduction compared to all other treatment groups (p = 0.0460) (Figure 6B).

A summary of all histology and immunohistologic changes can be seen in Figure 7.

## Discussion

Injury induced OA is a disorder affecting multiple organs of the synovial joint, yet many in vitro models to study injury induced OA only examine the cartilage. In this study, we sought to develop a co-culture model of injured cartilage and synoviocytes to determine if and how synoviocytes modulate the progression of an osteoarthritic phenotype in injured cartilage. The data presented here indicate that normal synoviocytes protect cartilage from the effects of injury by reducing aggrecanase gene expression, promoting collagen type 2 expression and reducing the incidence of injury induced focal cell loss and chondrocyte cluster formation.

The aggrecanases ADAMTS4 and 5 are the enzymes that are largely responsible for degrading aggrecan under pathologic conditions [26,27]. In this study when synoviocytes were cultured in the presence of injured cartilage there was a significant reduction in synoviocyte expression of both these aggrecanases, a change that suggest these cells respond to injured cartilage by down regulating catabolic activity. Matrix metalloproteinases are the endopeptidases that are primarily responsible for the degradation of collagens, while tissue inhibitor of metalloproteases inhibit MMP activity. Under normal conditions, MMPs and TIMPs exist in a state of dynamic equilibrium [28]. This balance is shifted under pathologic conditions to favor MMP expression and activity ultimately contributing to ECM degradation [9,29-33]. Synovial fluid from injured and osteoarthritic joints has been shown to contain high levels of MMP-1[34,35]. Contrary to the anabolic response of aggrecanase down regulation by synoviocytes in our study, we found an increased synoviocyte expression of MMP-1 when cultured in the presence of injured cartilage that was accompanied by a decrease in TIMP-1 expression which together suggests an increased catabolic activity by synoviocytes. Given that aggrecan is the major target molecule for ECM degradation, and that ADAMTS enzymes are more efficient at cleaving aggrecan then MMPs [36], it is possible that the down regulation of ADAMTS4 and 5 by synoviocytes may have a greater impact on cartilage degradation then the increased MMP-1 expression.

Although it is accepted that synovitis contributes to cartilage degradation and OA, we were unable to detect many of the putative pro-inflammatory cytokines, and the expression levels of those present in our study such as cox-2, TGFβ, IL-6 and FGF2 were unaffected by culture with cartilage. We were unable to detect IL-1β, IL-1RA, IL-10 or TNF-α mRNA in the synoviocytes via real time PCR. This could be explained by our synovial cell extraction and culture conditions favoring a more pure population of fibroblastic synoviocytes rather than synovial macrophages or that we used normal, un-inflamed synovial membrane. Synovial macrophages have been shown to have an important role in promoting inflammation as well as stimulating synovial fibroblasts to produce inflammatory cytokines [37]. Indeed in a study where macrophages were depleted from the population of cells digested from synovium explants, there was a significant reduction in the expression of inflammatory cytokines including IL-1 and MMPs [37]. Thus, the lack of IL-1β and TNF-α detection in the present study likely reflects the difference between using a purified population of fibroblastic synovial cells instead of synovium explants.

Interleukin -1β and MMP-13 have both been extensively demonstrated to play a prominent role in the progression of OA yet IL-1β gene expression by chondrocytes was not detected in the present study and MMP-13 expression levels were not affected by injury or culture condition. Interestingly this finding is consistent with a recent study conducted by Ross et al. 2012 involving an in vivo model of experimentally induced synovitis in the horse [38]. In the in vivo study the data strongly demonstrated the development of a model consistent with traumatic arthritis and OA, yet IL-1β gene expression levels were not consistently detectible and MMP-13 expression did not differ between normal and inflamed joints. These results collectively suggest IL-1β and MMP-13 gene expression may not be a critical component of traumatic joint injury in horses.

The differential expression of type 2 collagen and ADAMTS5 observed in injured cartilage cultured in the presence of synoviocytes compared to injured cartilage cultured alone further suggests a relationship exists between synoviocytes and injured cartilage. Type 2 collagen expression was the highest in injured cartilage when cultured in the presence of synoviocytes compared to any other cartilage condition tested. This unique finding suggests the presence of synoviocytes induces an anabolic response by chondrocytes that is specific to injured cartilage, as this increase was not seen in control cartilage cultured with synoviocytes. On the contrary, ADAMTS5 expression by chondrocytes of injured cartilage cultured in the presence of synoviocytes was higher than by chondrocytes of injured cartilage cultured alone. Again this effect of synoviocytes appears to be unique to injured cartilage as this increased expression was not observed in chondrocytes of cartilage in any other condition. In a similar study conducted by one of the co-authors (AJG), the culture of injured cartilage in the presence of synovium explants had a similar effect on chondrocyte gene expression [20]. In that Lee et al. study [20] the introduction of synovium explants to injured cartilage increased the expression of ADAMTS5 at the early time points after injury and the expression of ADAMTS4 was significantly increased for the duration of the experiment. In the Lee et al. study [20] study expression of type 2 collagen was not affected by the presence of synovium explants, as it was in the present study. Again, the results in the present study may highlight a response that is specific to the population of synovial fibroblasts that is lost when fibroblasts are grouped together with other synovial cells in the synovium.

The presence of synoviocytes also significantly increased TGFβ expression in injured cartilage at two days in culture when compared to any other time point or condition including baseline. Transforming growth factor β is an anabolic factor that induces chondrocyte synthesis of ECM molecules by mesenchymal stem cells and chondrocytes [39,40] and leads to increased chondrocyte proliferation [41]. Although the results seem contradictory, it is not uncommon for chondrocytes to express both anabolic and catabolic genes at one time, and may reflect an attempt of chondrocytes at cartilage repair and remodeling. In a study that tested repair tissue in an equine cartilage defect model, both normal and repair cartilage tested positive for the expression of genes associated with matrix synthesis and degradation [42].

Evaluating the gene expression data together the overall impact of synoviocytes on injured cartilage indicates synoviocytes promote a predominantly anabolic response in chondrocytes potentially to protect from typical degradation in response to injury.

Synoviocytes also had an impact on histologic changes in injured cartilage. Cartilage injury and OA have been associated with ECM degradation and ECM molecule loss [43-46]. In the present co-culture samples, we show that over time for all cartilage samples there was a steady decrease in SOFG staining which was used as a semi-quantitative evaluation of GAG content. Analysis of aggrecan content by IHC showed no significant differences between samples. In all samples there was a gradual reduction in type II collagen for the duration of the experiment resulting in a significant reduction at day thirty-two compared to day eight. Given this reduction occurred in all samples, one can assume it is likely an artifact of maintaining cartilage in static culture for thirty-two days. Taken together these data indicate the presence of synoviocytes did not impact ECM morphology.

Chondrocyte cell death is a typical result of cartilage injury and can contribute to the subsequent degradation of the ECM independent of mechanical trauma [47]. Chondrocyte cell death will occur either by necrosis or apoptosis (chondropoptosis). With necrosis the cells will swell and ultimately lyse. With apoptosis, the chondrocytes undergo programmed cell death which typically leads to the unique event of chondroptosis where autophagic vacuoles develop and digest the remaining cell fragments leaving an empty lacunae [48]. Cell death is believed to be an important consequence of injury contributing to pathologic changes in cartilage, such that hypocellularity and empty lacunae are parameters used to characterize osteoarthritic cartilage [49-51]. In these studies cell death was assessed by the two morphological parameters; first by identifying empty lacuna, and second by assessing focal regions with empty lacunae. In these experiments cell death was affected by duration in culture with the greatest incidence at thirty-two days in culture regardless of treatment. Additionally, all injured cartilage samples had a significantly greater incidence of cell death and focal cell loss when compared to control cartilage, and the increase in focal cell loss was significantly reduced in injured cartilage cultured with synoviocytes. The data presented here suggest synoviocytes protect injured cartilage from focal cell loss, a result that has not previously been reported in the literature.

As would be expected, chondrocyte clusters were detected in cartilage after injury [52,53]; however, when injured cartilage was cultured in the presence of synoviocytes, chondrocyte cluster formation was significantly reduced to a level that was no different than control cartilage. Furthermore, when injured cartilage was cultured in the presence of synoviocytes, chondrocyte cluster size was significantly smaller. Clusters have been shown to express markers of hypertrophy such as type X collagen [54], alkaline phosphatase [55] and osteocalcin [56], the matrix molecule fibronectin [57], and ECM degrading enzymes [58]. Synoviocytes appear to affect cluster formation and reduce the severity of this OA characteristic of cartilage in response to injury, which may have a significant impact on the progression of disease after injury.

## Conclusions

In summary, the data presented in this study collectively indicate that normal synoviocytes protect cartilage from the effects of cartilage injury, and reduce the progression of an OA phenotype. These data are consistent with a similar co-culture model of cartilage explants and synoviocytes [18]. Although it is widely accepted that the synovium releases pro-inflammatory molecules in response to joint injury, the data presented here indicate the catabolic effects of the synovium may not come from the fibroblastic synoviocytes and instead from other cells that make up the synovium such as macrophages, or alternatively inflamed synovial membrane has totally different characteristics than normal synovial membrane. Furthermore, the isolation and culture of the synoviocytes in this study is quite similar if not the same as that of isolating and expanding synovial derived stem cells [59,60]. In future studies, we will isolate synovial derived stem cells from the synovium to determine if those cells emulate the beneficial effects of synoviocytes in the present study.

## Abbreviations

OA: Osteoarthritis; ADAMTS4,5: Aggrecanase-1,2; MMP: Matrix metalloproteinase; TIMP: Tissue inhibitor of matrix metalloproteinase; PGE2: Prostaglandin E_2_; IL: Interleukin; TNF: Tumor necrosis factor; TGF: Transforming growth factor; FGF: Fibroblast growth factor; ECM: Extracellular matrix; GAPDH: Glyceradehyde 3-phosphate dehydrogenase; H&E: Hematoxylin and Eosin; SOFG: Safranin O fast green; GAG: Glycosaminoglycan; IHC: Immunohistochemistry.

## Competing interests

The authors declare that they have no competing interests.

## Authors’ contributions

CML participated in study deign, conducted all experiments and sample analysis. DDF conceived this study and participated in the design of the experiments, provided assistance for data analysis and statistical analysis and reviewed the manuscript. CWM participated in the development of the study design and review of the manuscript. JDK and AJL contributed to the technical development of the model used in the study and participated in manuscript review and revision. All authors read and approved the final manuscript.

## Pre-publication history

The pre-publication history for this paper can be accessed here:

http://www.biomedcentral.com/1471-2474/14/54/prepub
